# Surgical and Antimicrobial Treatment of Prosthetic Vascular Graft Infections at Different Surgical Sites: A Retrospective Study of Treatment Outcomes

**DOI:** 10.1371/journal.pone.0112947

**Published:** 2014-11-13

**Authors:** Stefan Erb, Jan A. Sidler, Luigia Elzi, Lorenz Gurke, Manuel Battegay, Andreas F. Widmer, Maja Weisser

**Affiliations:** 1 Division of Infectious Diseases and Hospital Epidemiology, University Hospital Basel, Basel, Switzerland; 2 Department of Vascular Surgery, University Hospital Basel, Basel, Switzerland; 3 Department of Clinical Research, University Hospital Basel, Basel, Switzerland; UNIFESP Federal University of São Paulo, Brazil

## Abstract

**Objective:**

Little is known about optimal management of prosthetic vascular graft infections, which are a rare but serious complication associated with graft implants. The goal of this study was to compare and characterize these infections with respect to the location of the graft and to identify factors associated with outcome.

**Methods:**

This was a retrospective study over more than a decade at a tertiary care university hospital that has an established multidisciplinary approach to treating graft infections. Cases of possible prosthetic vascular graft infection were identified from the hospital's infectious diseases database and evaluated against strict diagnostic criteria. Patients were divided into groups according to the locations of their grafts: thoracic-aortic, abdominal-aortic, or peripheral-arterial. Statistical analyses included evaluation of patient and infection characteristics, time to treatment failure, and factors associated specifically with cure rates in aortic graft infections. The primary endpoint was cure at one year after diagnosis of the infection.

**Results:**

Characterization of graft infections according to the graft location did show that these infections differ in terms of their characteristics and that the prognosis for treatment seems to be influenced by the location of the infection. Cure rate and all-cause mortality at one year were 87.5% and 12.5% in 24 patients with thoracic-aortic graft infections, 37.0% and 55.6% in 27 patients with abdominal-aortic graft infections, and 70.0% and 30.0% in 10 patients with peripheral-arterial graft infections. In uni- and multivariate analysis, the type of surgical intervention used in managing infections (graft retention versus graft replacement) did not affect primary outcome, whereas a rifampicin-based antimicrobial regimen was associated with a higher cure rate.

**Conclusions:**

We recommend that future prospective studies differentiate prosthetic vascular graft infections according to the location of the grafts and that rifampicin-based antimicrobial regimens be evaluated in clinical trials involving vascular graft infections caused by staphylococci.

## Introduction

Although a prosthetic vascular graft infection (PVGI) is a rare complication that occurs in 1–6% of patients after graft implantation, it is associated with a high mortality rate (up to 75%) and a major amputation rate that can be as high as 70% (range 1–70%) [1]–[5]. There is little available information with respect to optimal surgical and antimicrobial approaches to the management of PVGI. Current recommendations for treating PVGI are based mainly on small case series and expert opinion [6], [7].

The traditional approach to managing PVGI has been surgical removal of the infected prosthetic vascular graft with extra-anatomic bypass revascularization. Recently, alternative surgical approaches such as in situ graft replacement [1], [8]–[16] or graft retention with thorough debridement have been more commonly used, in particular in patients with thoracic-aortic and peripheral-arterial PVGI [6], [17], [18]. In the published literature to date, removal and replacement of the graft is still considered the standard surgical approach in most patients with PVGI [19]–[25]. Unfortunately, prospective studies that compare the various surgical treatment strategies are lacking [6], [7]. Antimicrobial therapy, as an essential adjunct to the surgical management of an infection, also has not been studied systematically. The duration of antimicrobial therapy can range from 11 days to more than a year or even to lifelong suppressive therapy [26]–[28].

The goal of this retrospective study was to compare and characterize these infections with respect to the location of the grafts and to find treatment factors associated with a beneficial clinical outcome. The overall goal of the study was achieved in terms of establishing, that the location of a graft does influence the characteristics and treatment outcomes for PVGI, and that rifampicin-based antimicrobial regimens might be independently associated with a higher cure rate.

## Methods

### Study Population and Study Conduct

The setting for this study was a 700-bed tertiary care university hospital in northern Switzerland that serves a regional population of approximately 400,000. Interdisciplinary management of patients with a PVGI is standard practice at this hospital: an infectious disease specialist recommends the appropriate antimicrobial treatment, and the attending surgeon determines the need for and type of appropriate surgical intervention. In this study, we retrospectively analyzed PVGI treatment methods and outcomes over a period of almost 12 years, from January 1, 2001, through August 2012, for all patients ≥18 years of age who had been diagnosed with an aortic PVGI or a peripheral-arterial PVGI. Cases of PVGI were identified from entries in the hospital's infectious diseases database. Then related clinical and laboratory data were extracted from the hospital's electronic and written medical records and independently assessed for completeness by an infectious disease specialist. If follow-up data were missing for patients, either the patient or the mentoring doctor was contacted by telephone to obtain the necessary information. We excluded from the study patients whose infections did not meet the study definition of a PVGI, patients for whom outcome data were insufficient, and patients who had hemodialysis-shunt graft infections. The primary endpoint was a cure at one year after diagnosis of the PVGI. Secondary endpoints were a cure by the conclusion of antimicrobial treatment, all-cause and graft-related early mortality (≤30 days after diagnosis of the infection), and all-cause and graft-related mortality ≤1 year after diagnosis of the PVGI.

The cantonal Ethics Committee of Basel (EKBB), Switzerland, approved this study (number 263/11). No informed consent had to be given by participants for their clinical records to be used in this retrospective study. Patient records were anonymized and de-identified prior to analysis.

### Definitions

In the absence of an internationally accepted definition for a PVGI, we adapted the Centers for Disease Control and Prevention (CDC) definitions of organ-space surgical site infections corresponding to Group 3 of the Szilagyi classification and the definitions by Legout et al. [4], [29], [30]. We considered a patient to have a PVGI if, at a minimum, one of the following criteria were met: (a) the patient had a microorganism isolated that was aseptically obtained from a biopsy or swab of the immediate area around the graft, (b) there was histopathological or radiological evidence of an infection involving the graft and the surrounding tissue, or (c) the patient had continuous bacteremia in the presence of a graft with no other apparent focus of infection. Continuous bacteremia was defined as ≥2 blood cultures with the same pathogen obtained at different times. When potential contaminants such as coagulase-negative staphylococci or *Propionibacterium acnes* were present, the infection had to meet either criterion (a) or criterion (b).

Aortic PVGI were divided into two groups: infections of the ascending or descending thoracic aorta and infections of the abdominal aorta with or without iliac-femoral involvement. Peripheral PVGI were defined as infections affecting infra-inguinal arterial grafts.

Cure was defined as the absence of clinical signs of infection (temperature ≤38.5°C; and in the case of a peripheral-arterial graft, no local inflammation), normal graft function (no infection-related bleeding, ischemia distal of the graft or major amputation), the absence of radiological evidence of infection (no fluid or air collection around the graft or other evidence of infection as determined by a radiologist), normal levels of inflammation parameters (C-reactive protein ≤10 mg/l and a white-blood cell count of 4−12×10^9^/l). Treatment failure was defined as the presence of any of the above-mentioned signs of infection without an obvious alternative explanation for them (e.g., increase in inflammation parameters after being in the normal range due to a urinary tract infection at follow-up).

Empirical antibiotic therapy was defined as the first antibiotic given with suspicion of infection. Targeted therapy was defined as antibiotic treatment directed against the isolated microorganisms following susceptibility testing. Adequate therapy was defined as any antibiotic treatment efficacious against the detected organism according to susceptibility testing.

### Statistical Analysis

Basic socio-demographic characteristics, concurrent medical conditions, surgical procedures, and antimicrobial treatments were compared using the Chi-Square or Fisher's exact test for categorical variables and the Mann-Whitney test for continuous variables. Cox regression was used to investigate factors associated with outcomes at one year after diagnosis of the infection in patients who had an aortic PVGI. The time to treatment failure was determined by using Kaplan-Meier estimates and the groups were compared using the log-rank test. The statistical software STATA version 11 (StataCorp LP, Texas, USA) was used for all analyses. A p-value ≤0.050 was considered significant.

## Results

### Patient's Characteristics and Clinical Presentations

**Figure 1:**
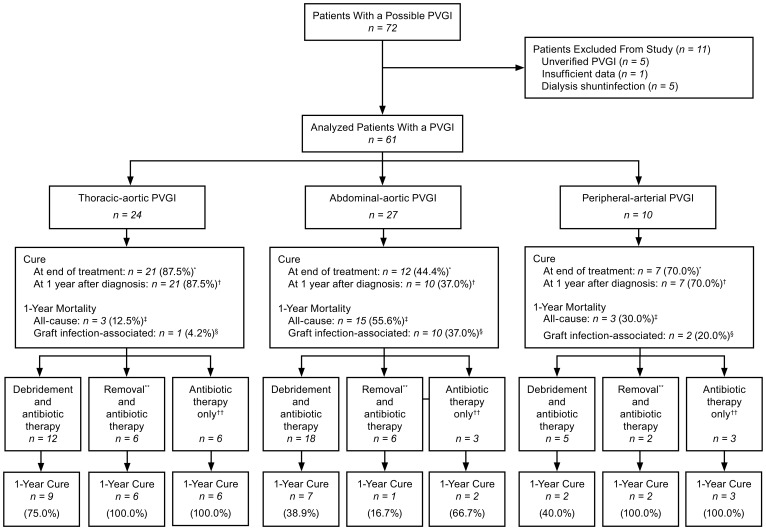
Comparison of primary and secondary outcomes in the treatment of prosthetic vascular graft infection. PVGI  =  prosthetic vascular graft infection. ^*^p-value  = 0.006. ^†^p-value  = 0.001. ^‡^p-value  = 0.004. ^§^p-value  = 0.012. **Removal or replacement of the infected vascular graft. ^††^No surgical intervention; antimicrobial treatment only.

During the time period covered by the study, 72 patients were identified in the infectious diseases database as possibly having a PVGI (Figure 1). Of these, a total of 11 patients were excluded from the study, 5 because their infections did not meet the study criteria for a PVGI, 1 because there was insufficient outcome data, and 5 because they had hemodialysis-shunt graft infection. The remaining 61 patients with a confirmed PVGI were included in the analysis. In 24 of the analyzed 61 patients, the site of the infection was a thoracic graft (18 of whom with a composite graft and heart valve replacement); in 27 patients, the site of the infection was an abdominal graft (21 an aorto-iliac graft, 6 a graft confined to the abdominal aorta); and in 10 patients, the site of the infection was a peripheral graft. Two patients had an endoluminal graft replacement (1 of them a thoracic graft, and 1 of them an abdominal-iliac graft). In 47 patients, the reason for initially implanting a prosthetic vascular graft was an aneurysm (46 aortic grafts; 1 peripheral graft). In 11 patients, the reason for the graft implant was an occlusive arterial disease (3 aortic grafts; 8 peripheral grafts). In the remaining three patients, the reason for the graft implant was an abscess, a paravalvular leak in another patient and a severe aortic stenosis in the third patient.

There were some statistically significant differences between the groups of patients (Table 1). Patients with a thoracic PVGI were younger (median age 58 years, interquartile range [IQR] 48–65) and were less likely to have a concurrent cardiovascular disease (45.8%) than patients with an abdominal or a peripheral PVGI (p<0.001 and p = 0.007, respectively). Patients with an abdominal PVGI had more often required an early revision (<24 hours after primary graft implantation) of the original graft (37.0%) than had patients with a thoracic or a peripheral PVGI (8.3% and 10.0%, respectively; p = 0.034).

**Table 1:** Comparison of patient characteristics and clinical presentation with respect to graft location in patients with prosthetic vascular graft infection.*

Variable	Thoracic-Aortic PVGI *(n = 24)*	Abdominal-Aortic PVGI *(n = 27)*	Peripheral-Arterial PVGI *(n = 10)*	p-Value†
**Patient Characteristics at the Time of Graft Infection**				
Median age in years	58 (IQR 48–65)	70 (IQR 66–76)	74 (IQR 57–78)	<0.001
Male patients, n	21 (87.5%)	25 (92.6%)	7 (70.0%)	0.202
Median BMI in kg/m^2^	27 (IQR 24–28)	25 (IQR 23–28)	25 (IQR 20–27)	0.622
Patients with a ICU stay, n	9 (37.5%)	11 (40.7%)	3 (30.0%)	0.938
Cardiovascular disease‡, n	11 (45.8%)	18 (66.7%)	10 (100%)	0.007
Diabetes mellitus, n	4 (16.7%)	5 (18.5%)	1 (10.0%)	0.823
COPD, n	4 (16.7%)	8 (29.6%)	3 (30.0%)	0.533
Renal impairment§, n	5 (20.8%)	13 (48.1%)	1 (10.0%)	0.037
**Complications at the Time of Graft Implantation**				
Open graft implantation, n patients	23 (95.8%)	26 (96.3%)	10 (100%)	0.813
Emergency surgery, n patients	5 (20.8%)	7 (25.9%)	3 (30.0%)	0.791
Early graft revision <24 hours, n patients	2 (8.3%)	10 (37.0%)	1 (10.0%)	0.034
Blood transfusions, n patients	9 (37.5%)	10 (37.0%)	2 (20.0%)	0.631
Intubation >24 hours, n patients	5 (20.8%)	4 (14.8%)	0 (0.0%)	0.399
**Onset of Graft Infection**				
Median time from graft implant to diagnosis of infection in days	37 (IQR 11–131)	40 (IQR 6–440)	15 (IQR 6–26)	0.538
Early onset of infection (≤4 months after graft implant), n patients	18 (75.0%)	15 (55.6%)	8 (80.0%)	0.384
Late onset of infection (>4 months after graft implant), n patients	6 (25.0%)	12 (44.4%)	2 (20.0%)	0.384
**Signs and Symptoms of Graft Infection**				
Fever >38.5°C, n patients	12 (50.0%)	12 (44.4%)	3 (30.0%)	0.620
Erythema, n patients	3 (12.5%)	1 (3.7%)	5 (50.0%)	0.003
Wound discharge, n patients	5 (20.8%)	4 (14.8%)	3 (30.0%)	0.521
Bleeding, n patients	1 (4.2%)	2 (7.4%)	1 (10.0%)	0.802
Ischemia, n patients	1 (4.2%)	4 (14.8%)	4 (40.0%)	0.029
Severe sepsis, n patients	1 (4.2%)	4 (14.8%)	0 (0.0%)	0.372
Median white cell count as n×10^9^/l	11.3 (IQR 9.4–19.5)	11.9 (IQR 8.3–23.5)	9.9 (IQR 8.2–18.2)	0.918
Median C-reactive protein in mg/l	102 (IQR 53–174)	172 (IQR 107–261)	95 (IQR 47–198)	0.042
A CT scan that showed fluid or air around the graft prosthesis, n patients	20 (83.3%)	13 (48.2%)	2 (20.0%)	0.001
A positive blood culture**, n patients	15 (62.5%)	13 (48.1%)	3 (30.0%)	0.210
A positive tissue culture††, n patients	14 (58.3%)	24 (88.9%)	10 (100%)	0.040
**Microbiological Characteristics: Patients with a identified microorganism** ‡‡				
*Staphylococcus aureus* §§, n	4 (16.7%)	6 (22.2%)	4 (40.0%)	0.362
Coagulase-negative staphylococci, n	8 (33.3%)	3 (11.1%)	5 (50.0%)	0.030
*Propionibacterium acnes*, n	5 (20.8%)	0 (0.0%)	0 (0.0%)	0.015
*Enterococcus* spp., n	5 (20.8%)	11 (40.7%)	2 (20.0%)	0.257
*Escherichia coli*, n	0 (0.0%)	4 (14.8%)	0 (0.0%)	0.068
*Pseudomonas aeruginosa*, n	1 (4.2%)	1 (3.7%)	1 (10.0%)	0.717
*Enterobacter* spp., n	1 (4.2%)	8 (29.6%)	1 (10.0%)	0.041
*Proteus mirabilis*, n	1 (4.2%)	1 (3.7%)	0 (0.0%)	0.813
*Klebsiella* spp., n	0 (0.0%)	2 (7.4%)	0 (0.0%)	0.272
*Stenotrophomonas maltophilia*, n	0 (0.0%)	2 (7.4%)	0 (0.0%)	0.272
*Serratia marcescens*, n	0 (0.0%)	0 (0.0%)	1 (10.0%)	0.075
*Morganella morganii*, n	0 (0.0%)	1 (3.7%)	0 (0.0%)	0.527
Anaerobic bacteria, n	2 (8.3%)	10 (37.0%)	1 (10.0%)	0.243
*Candida* spp., n	1 (4.2%)	10 (37.0%)	0 (0.0%)	0.003
Patients with polymicrobial infections, n	5 (20.8%)	18 (66.7%)	4 (40.0%)	0.004

PVGI  =  prosthetic vascular graft infection; IQR  =  interquartile range; BMI  =  body mass index; COPD  =  chronic obstructive pulmonary disease; ICU  =  intensive care unit; CT  =  computed tomography.

*Percentages and IQR values indicate relationship to the total number of patients within each respective group, not to the total number of patients included in the study.

† A p-value ≤0.050 was considered significant.

‡ Defined as the total number of patients with a medical history of peripheral arterial occlusive disease, coronary heart disease, or cerebrovascular disease.

§ Defined as an estimated glomerular filtration rate <60 ml/min/1.73 m^2^ at the time of PVGI diagnosis.

**There had been no blood cultures drawn for three patients with a thoracic PVGI and for three patients with a peripheral PVGI.

†† Defined as swabs or biopsies taken from the infected peri-graft areas whose cultures showed microbial growth.

‡‡ Numbers (%) refer to patients with a specific pathogen isolated; multiple microorganisms per patient were possible. The causative microorganism was identified in blood cultures or in tissue cultures from surgical site for 60 (98.4%) of the 61 patients in the study.

§§ One out of 14 *Staphylococcus aureus* isolates was methicillin-resistant.

The time period until development of graft infection after implantation typically was <2 months. At the time of diagnosis, 44.3% of all patients had a fever >38.5°C. Local signs of infection were reported most often in patients with peripheral PVGI (erythema in 50.0% and ischemia in 40.0% of these patients). Wound discharge was reported in 19.7% of all patients. The median C-reactive protein level for all patients was 143 mg/l (IQR 78–239).

In 12 out of 35 patients with periprosthetic fluid or air detected on a computed tomography scan, the examination was performed >2 months after primary graft implantation. Additionally, in 5 patients with a thoracic PVGI an echocardiography was suggestive for a PVGI 8–52 days after primary graft implantation. Four patients with an abdominal PVGI had an aorto-enteric fistula; one patient with a thoracic PVGI presented with an aorto-bronchial fistula.

### Microbiological Data

The infecting microorganism was identified in 60 (98.4%) of the 61 patients in the study (Table 1). Of these 60 patients, 48 (80.0%) had one or more swabs or biopsies from the infected peri-graft area that documented microbial growth, 19 of whom also had positive blood cultures with the same microorganism. In 12 patients (9 of whom had thoracic PVGI), a positive blood culture was the only microbiological evidence of infection. In one patient for whom there was no microbiological confirmation of infection, the diagnosis of a thoracic PVGI was based on clinical signs of infection and radiological evidence from a series of positron emission tomography–computed tomography (PET-CT) scans that showed persistent fluid collection and hypermetabolism around the graft.

The frequency with which any particular causative microbiological agent was associated with a PVGI varied according to the location of the graft. Staphylococci were predominant in thoracic and peripheral PVGI. Of note, only one out of 14 *Staphylococcus aureus* isolates was methicillin-resistant in patients with a PVGI. Polymicrobial infections were frequently identified in patients with abdominal PVGI (66.7%), comprising mainly gram-negative and anaerobic bacteria, enterococci and *Candida* spp.

### Surgical and Antimicrobial Therapy

Surgical intervention was used to treat PVGI in 49 (80.3%) of the patients (Table 2). Of these, 32 patients (65.3%) required two or more operations. The most common technique was debridement with graft retention (35 patients, 57.4%). Primary graft removal or replacement was used in 14 patients (22.9%). In four of the 14 patients for whom the original graft was removed, the surgery also involved an extra-anatomical bypass. Two of these were patients with abdominal PVGI (7.4% of all abdominal graft infections), and two were patients with peripheral PVGI (20.0% of all peripheral graft infections). In the ten patients with a in situ graft replacement, either cryopreserved homografts or Dacron grafts were used—cryopreserved homografts in two patients with thoracic PVGI and in three patients with abdominal PVGI, Dacron grafts in four patients with thoracic PVGI and in one patient with an abdominal PVGI. Additional vacuum-assisted local therapy was used in 16 patients (26.2%) and plastic surgery with a muscle flap in 7 patients (11.5%).

**Table 2:** Comparison of therapies for prosthetic vascular graft infections with respect to the location of the graft.*

Variable	Thoracic-Aortic PVGI *(n = 24)*	Abdominal-Aortic PVGI *(n = 27)*	Peripheral-Arterial PVGI *(n = 10)*	p-Value†
**Graft Infection Therapies**				
Graft removal or replacement and antimicrobial therapy, n patients	6 (25.0%)	6 (22.2%)	2 (20.0%)	0.588
Debridement with graft retention and antimicrobial therapy, n patients	12 (50.0%)	18 (66.7%)	5 (50.0%)	
Antimicrobial therapy only, n patients	6 (25.0%)	3 (11.1%)	3 (30.0%)	
**Surgical Therapies**				
Median number of surgical revisions per patient‡	1 (range 1–5)	2 (range 1–10)	2 (range 1–6)	0.193
Vacuum-assisted closure§, n patients	5 (20.8%)	9 (33.3%)	2 (20.0%)	0.630
Plastic surgery with muscle flap, n patients	2 (8.3%)	3 (11.1%)	2 (20.0%)	0.668
**Empirical Antimicrobial Therapies**				
Amoxicillin-clavulanate or flucloxacillin, n patients	8 (33.3%)	6 (22.2%)	7 (70.0%)	0.022
Piperacillin-tazobactam or carbapenem, n patients	9 (37.5%)	19 (70.4%)	2 (20.0%)	
Ceftriaxone, n patients	3 (12.5%)	1 (3.7%)	1 (10.0%)	
Vancomycin, n patients	4 (16.7%)	1 (3.7%)	0 (0.0%)	
Adequate empirical therapy**, n patients	24 (100%)	26 (96.3%)	9 (90.0%)	0.303
**Biofilm-active Antimicrobial Therapies**				
Rifampicin-based, n patients	16 (66.7%)	5 (18.5%)	7 (70.0%)	0.001
Ciprofloxacin-based, n patients	8 (33.3%)	10 (37.0%)	4 (40.0%)	0.940
**Antimicrobial Therapy in Patients Alive at One Year after Diagnosis**				
Median duration in days of antimicrobial therapy††	92 (IQR 67–175)	91 (IQR 52–246)	46 (IQR 39–76)	0.023
Patients who switched from an intravenous antimicrobial regimen to an oral antimicrobial regimen‡‡, n	11 (52.4%)	5 (41.7%)	5 (71.4%)	0.531
Median time in days between start of intravenous antimicrobial regimen and the switch to an oral regimen	42 (IQR 13–58)	34 (IQR 21–52)	19 (IQR 15–37)	0.320

PVGI  =  prosthetic vascular graft infection; IQR  =  interquartile range

*Percentages, IQR values, and ranges indicate relationship to the total number of patients within each respective group, not to the total number of patients included in the study.

† A p-value ≤0.050 was considered significant.

‡ In patients with at least one surgical revision of their graft.

§ Application of negative pressure to the local wound environment using a sealed foam dressing connected to a vacuum pump.

**Adequate empirical therapy according to the susceptibility testing of the respective isolated pathogen.

†† Total duration of both empirical and pathogen-specific antimicrobial therapy.

‡‡ Percentages apply to the total number of patients who were alive at one year within each respective group.

At one year after diagnosis among patients who had peripheral PVGI, there had been one patient with infection-related graft failure (limb ischemia without need for amputation) and three patients (30.0%) for whom amputation had been necessary, two of them requiring limb (major) amputation and the third a less radical (minor) amputation.

All 61 patients received antimicrobial therapy (Table 2). Of these, 12 (19.7%) had not had any surgical intervention and were treated only with antibiotics. Empirical antimicrobial therapy was adequate in 96.7% and consisted of a beta-lactam antibiotic in 56 patients (91.8%). In the 49 patients with a combined medico-surgical treatment, the median time interval from start of empirical antimicrobial therapy to surgical graft revision was 0.0 days (IQR -1.0–5.5 days). The time interval from initiation of empirical antimicrobial therapy to surgical graft revision did not differ significantly between the 2 surgical treatment groups (p = 0.440; debridement versus graft removal/replacement).

For the targeted treatment, a biofilm-active antibiotic treatment was given whenever possible: A rifampicin-based combination was used in 45.9% of the 61 patients for treating mainly *Staphylococcus*-related infections. A ciprofloxacin-based regimen was used in 36.1% of the 61 patients for treating gram-negative infections.

All ten patients who had received antibiotic treatment for more than six months were cured at one year after diagnosis of the infection (5 cases of thoracic PVGI, 4 cases of abdominal PVGI, 1 case of a peripheral PVGI). The reason for prolonged treatment in 60.0% of these patients was that the infection was a polymicrobial in nature, often including *Candida* spp. (40%), and a complicated treatment course with several hospitalizations (70%). The 33 patients who had aortic PVGI and had survived to at least one year after diagnosis of the infection had received antimicrobial therapy for a median total duration of 92 days (IQR 60–191). Eight of these patients had not had any surgical intervention and had been treated with antibiotics only. For them, the median total duration of antimicrobial therapy was prolonged (171 days; IQR 64–278). After completion of an antimicrobial therapy regimen, treatment failed in only three of patients, all of them with an abdominal PVGI.

### Outcome

The overall one-year cure rate in the study was 62.3% (Figure 1). The treatment outcome varied according to the location of the graft (p = 0.001), the best outcomes occurring in patients with thoracic PVGI (a one-year cure rate of 87.5%), followed by patients with peripheral PVGI (70.0%) and then by patients with abdominal PVGI (37.0%). All-cause 30-day mortality among patients with abdominal PVGI was 33.3%, and their all-cause mortality at one-year after diagnosis of an infection was 55.6%. In patients with thoracic PVGI, all-cause 30-day mortality was 4.2% and all-cause mortality at one year was 12.5%. In patients with peripheral PVGI, all-cause 30-day mortality was 0.0% and all-cause mortality at one year was 30.0%.

**Figure 2:**
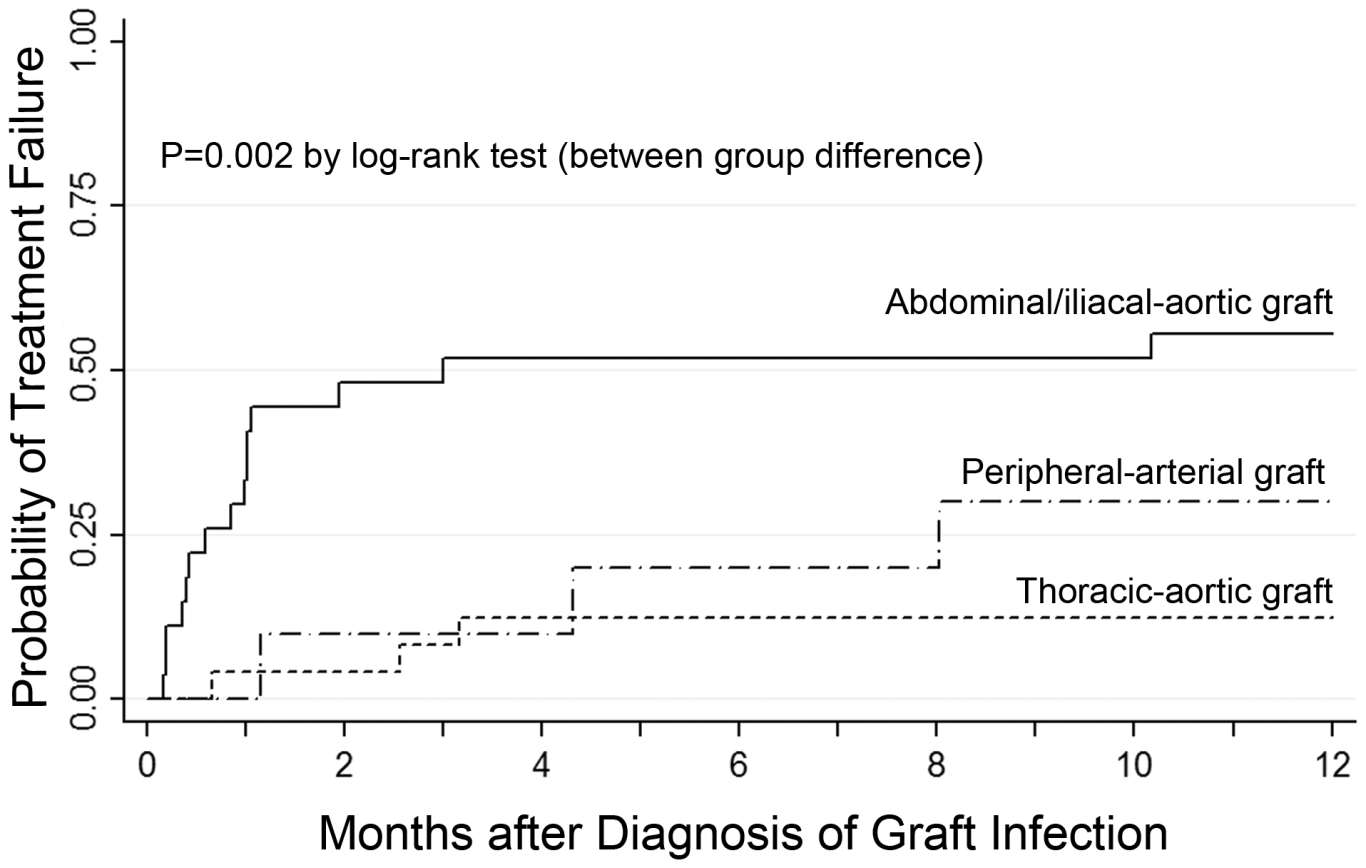
Comparison of time to treatment failure in different types of prosthetic vascular graft infections. Infections are compared with respect to the location of the prosthetic vascular graft using Kaplan-Meier estimates.

Treatment failure usually occurred within the first 3 months after diagnosis of the infection, and in case of an abdominal PVGI, frequently happened within the first month after diagnosis (Figure 2). When treatment for a PVGI failed, it was always fatal except in the case of two patients with abdominal PVGI who survived, one with a persistent abscess and one with an aorto-duodenal fistula.

Uni- and multivariate analyses of variables in this study with respect to all 51 aortic PVGI (both thoracic and abdominal) and the related outcomes (Table 3) show that increasing age and abdominal PVGI with iliac-femoral graft involvement were associated with a poor outcome (hazard ratio [HR] 0.33; 95% CI 0.15–0.74; [p = 0.007] and HR 0.07; 95% CI 0.02–0.32; [p = 0.001], respectively), that neither graft retention (HR 0.46; 95% CI 0.14–1.50; p = 0.20) nor graft replacement (HR 0.87; 95% CI 0.23–3.26; p = 0.84) was associated with treatment failure. The only factor positively associated with a higher cure rate in uni- and multivariate analysis was treatment with a rifampicin-based antimicrobial regimen in patients with aortic PVGI (in multivariate analysis; HR 6.88; 95% CI 1.33–35.4; p = 0.021).

**Table 3:** Predictors of cure at one year in 51 patients with aortic prosthetic vascular graft infections.*

Variable	Univariate Analysis			Multivariate Analysis†		
	HR	95% CI	p-Value‡	HR	95% CI	p-Value‡
Age§	0.33	0.15–0.74	0.007	0.45	0.19–1.11	0.083
Female gender	0.96	0.15–6.35	0.970	—	—	—
**Location of the Graft**						
Thoracic	1	—	—	—	—	—
Abdominal, with noiliac-femoral graft involvement	0.14	0.02–1.06	0.057	—	—	—
Abdominal withiliac-femoral graft involvement	0.07	0.02–0.32	0.001	0.24	0.05–1.16	0.076
Intubation >24 hours	0.44	0.10–1.91	0.276	—	—	—
Early surgical graft revision <24 hours after implantation	1.30	0.42–4.03	0.645	—	—	—
Polymicrobial infection	0.72	0.23–2.24	0.573	1.93	0.41–9.13	0.405
**Type of Surgical Treatment**						
Graft retention and debridement	0.46	0.14–1.50	0.196	—	—	—
Graft replacement	0.87	0.23–3.26	0.842	0.89	0.15–5.31	0.899
**Local Surgical Therapy Associated with Graft Revisions/Replacements**						
Vacuum-assisted closure	0.54	0.16–1.88	0.335	—	—	—
Plastic surgery	0.96	0.15–6.35	0.970	—	—	—
**Antimicrobial Therapy**						
Antimicrobial regimen that included rifampicin	7.85	1.90–32.45	0.004	6.88	1.33–35.4	0.021

HR  =  hazard ratio; CI  =  confidence interval; PVGI  =  prosthetic vascular graft infection.

*Aortic prosthetic vascular graft infections diagnosed in 51 patients in this single-center study of PVGI over a period of almost 12 years.

† Multivariate analysis adjusted for age of patient, location of the aortic graft, graft replacement, presence of a polymicrobial infection, and the use of rifampicin in the antibiotic regimen.

‡ A p-value ≤0.050 was considered significant.

§ Each 10-year increment of increase in age is associated with a 0.33 risk of less favorable treatment outcome.

## Discussion

Analysis of graft infections according to the locations of the associated grafts showed that these infections differ in terms of their clinical, microbiological and outcome characteristics. Patients who underwent graft-preserving surgery did not seem to have a worse outcome than patients whose graft was removed. Our data have to be interpreted with caution because of the heterogeneity of the patient population and the retrospective design of the study. However, another recent prospective cohort study did also find that in 54 patients with aortic PVGI there was no difference in outcome with respect to vascular graft removal versus graft preservation (in-hospital mortality was 22.2% in both groups) [4]. It's also been reported that graft-preserving strategies have proven successful in recent studies, especially in patients with poor physiologic reserve, composite valve graft infections, and a sternal infection with secondary involvement of the graft [17], [31], [32]. In contrast, Maze et al. reported a high mortality rate of 59% (median 40 months) in 17 patients with abdominal PVGI treated with graft retention [33].

The one-year all-cause 35.3% mortality rate for patients with aortic PVGI in our study corresponds to other published mortality rates of 15–59% for this type of infection [19]–[24], [33], although many of the other studies were quite small, provided mostly in-hospital mortality rates, and did not distinguish between the different locations of the PVGI. This study shows that patients with abdominal PVGI in particular tended to have unfavorable long-term outcomes, and patients with thoracic PVGI tended to have significantly better outcomes. This observation can be partly explained by the fact that patients with abdominal PVGI frequently had renal impairment (48.1%), which has been shown to be a predictor of treatment failure in PVGI [34]. In addition, abdominal PVGI was often the result of an intra-abdominal, polymicrobial infection (e.g. due to bowel ischemia and perforation), whereas thoracic PVGI was frequently a result of a surgical site infection caused by less virulent skin bacteria such as coagulase-negative staphylococci. As a consequence, empirical antimicrobial treatment for thoracic PVGI should cover mainly gram-positive bacteria, whereas in the case of abdominal PVGI broad-spectrum antibiotics covering gram-negative and anaerobic bacteria are mandatory.

A conservative treatment plan with antimicrobial treatment only and no surgical intervention was used in 9 of the patients with an aortic PVGI (17.6%). Of these patients, 5 had a thoracic composite valve PVGI that was caused by a low virulent pathogen (coagulase-negative staphylococci or propionibacteria) and had received prolonged antimicrobial therapy over a period of >6 months, leading to a successful cure rate of 90%. This outcome differs from other studies in which a treatment strategy without surgery was reported to be a risk factor for failure in aortic PVGI [35] and might reflect a difference in how patients were selected for surgery or antimicrobial treatment regimens.

Most treatment failure in patients with aortic PVGI in this study occurred within the first three months after diagnosis of the infection. This may mean that prolonged antibiotic treatment for more than three months or as lifelong suppressive therapy might not actually be necessary on a routine basis for all patients as had been suggested in other studies [26]–[28]. The one therapy found to be positively associated with a better outcome in this study was the administration of a rifampicin-based antimicrobial regimen in patients with aortic PVGI. The benefit of rifampicin might be relevant primarily in biofilm-producing gram-positive pathogens, as has been shown to be the case for prosthetic joint infections [36], [37]. Both in vitro studies and animal studies have shown rifampicin-coated grafts to be effective in the treatment (in situ replacement) and prevention of a PVGI, but results of clinical studies have been inconclusive [1], [12], [38]–[45]. Legout et al. demonstrated in a recent observational study a potential benefit of rifampicin-containing regimens in staphylococcal PVGI [34]. In our population, only one out of 14 *Staphylococcus aureus* isolates was methicillin-resistant. Outcome might be worse in populations with a high rate of PVGI caused by *S. aureus*, especially methicillin-resistant *S. aureus*, as has been previously shown for patients with infra-inguinal PVGI [46].

The strength of this study is that to the best of our knowledge it is the first large series that uses strict diagnostic criteria and compares the outcomes with respect to different surgical and antibiotic treatment modalities for this rare infectious complication. Limitations of the study are due to its retrospective design. First, direct comparison of the different surgical and antimicrobial treatment strategies has to be interpreted with caution because the patient groups were small as well as heterogeneous. Second, surgical management might have changed over the study period with a tendency toward more graft-preserving techniques. Third, the small sample size for patients who had been diagnosed with peripheral PVGI prevented adequate statistical analysis of this group.

In conclusion, abdominal-aortic, thoracic-aortic, and peripheral PVGI are three quite distinct clinical entities and the prognosis for treatment of a PVGI seems to be influenced by the location of the PVGI. Therefore, we are recommending that future prospective studies focus on analyzing PVGI on a site-specific basis and that rifampicin-based antimicrobial regimens be evaluated in clinical trials involving graft infections caused by staphylococci.
